# Antibiotics are not needed during tube thoracostomy for spontaneous pneumothorax: an observational case study

**DOI:** 10.1186/1749-8090-1-43

**Published:** 2006-11-13

**Authors:** Guven Olgac, Umit Aydogmus, Lutfiye Mulazimoglu, Cemal Asim Kutlu

**Affiliations:** 1 Department of Thoracic Surgery, Sureyyapasa Chest and Cardiovascular Diseases Teaching and Research Hospital, Istanbul, Turkey; 2 Department of Thoracic Surgery, Yedikule Teaching and Research Hospital for Chest Diseases and Thoracic Surgery, Istanbul, Turkey; 3 Department of Infectious Diseases and Clinical Microbiology, Marmara University School of Medicine, Istanbul, Turkey

## Abstract

**Background:**

Usefulness of prophylactic antibiotics following tube thoracostomy remains controversial in the literature. In this study, we aimed to investigate the consequences of closed tube thoracostomy for primary spontaneous pneumothorax without the use of antibiotics.

**Methods:**

One-hundred and nineteen patients underwent tube thoracostomy for primary spontaneous pneumothorax. None of them received prophylactic antibiotic treatment. Eight patients with prolonged air leak undergoing either video assisted thoracoscopic surgery or thoracotomy were excluded.

**Results:**

Of the remaining 111 (104 male and 7 female), 28 (25%) patients developed some induration around the entry site of chest tube that settled without further treatment. White blood cell count was high without any other evidence of infection in 12 (11%) patients and returned to its normal levels before discharge home in all. There was also some degree of fever not lasting for more than 48 hours in 8 (7%) patients. Bacterial cultures from suspected sites did not reveal any significant growth in these patients.

**Conclusion:**

Prophylactic antibiotic treatment seems avoidable during closed tube thoracostomy for primary spontaneous pneumothorax. This policy was not only cost-effective but also prevented our patients from detrimental properties of unnecessary antibiotic use, such as development of drug resistance and undesirable side effects.

## Background

Primary spontaneous pneumothorax (PSP) usually occurs following rupture of a subpleural bleb or bulla located at apices of the lungs without an associated underlying pulmonary disease. It is most frequently seen in tall, smoker and young adults with a male to female ratio of 6:1 [1,2]. Evacuation of the pleural air with simple aspiration or closed tube thoracostomy (CTT) is the treatment of choice in most cases. More invasive surgical procedures such as bullectomy and/or pleurodesis with either pleurectomy or pleural abrasion are usually recommended in cases with recurrent disease and occasionally at the first episode in certain circumstances [1].

Different opinions exist regarding the use of prophylactic antibiotics following insertion of a chest tube and only very few and rather old papers on this topic are listed in recent literature [3,4]. BTS (British Thoracic Society) guidelines for the management of spontaneous pneumothorax either do not examine or give indications on antibiotics use. Attention was pointed only toward the importance of sterile technique in CTT [1]. Antibiotics are not obligatory for the prophylaxis of surgical infections in the current practice. It may therefore be omitted in cases with no additional risk factors necessitating antibiotic prophylaxis following CTT [5]. Thus, we have adopted our practice accordingly, and have not been using prophylactic antibiotics in such cases since the beginning of the year 2001. This study aims to rationalize the use of prophylactic antibiotics in this cohort of patients and compare our three and a half years of experience with those of reported in the medical literature.

## Methods

One hundred and nineteen patients underwent CTT for PSP between January 2001 and June 2004. Eight patients with prolonged air leak (> 7 days) undergoing either video-assisted thoracoscopic surgery or thoracotomy were started on our standard prophylactic antibiotic treatment [6] and therefore excluded from the study. Data of the remaining 111 patients was extracted from hospital's case notes and evaluated retrospectively.

Diagnosis of a PSP was achieved on the basis of postero-anterior chest X-ray (CXR) together with detailed questioning of patient's past medical history. A computerized tomography of the chest was also obtained for patients in whom the diagnosis was unconvinced. Patients with secondary spontaneous pneumothorax and those of PSP in association with either history of a malignancy or a systemic disease (e.g. diabetes mellitus, steroid use, chemotherapy etc.) were excluded from the study.

CTT was performed in a sterile setting in the emergency room using 10% povidone iodine for skin antisepsis and 2% Lidocaine for local anaesthesia. According to patient's body size, chest tubes of either 20 or 24 Fr size were inserted via 5th or 6th intercostal space at the mid-axillary line of the affected site, fixed to the skin with No. 0 silk suture and connected to an under-water seal drainage system. Position of the drain and lung expansion was verified with a control CXR that was obtained within one hour following the procedure. If the lung was not fully expanded, a suction of 20 cm H_2_O was applied on the drain. Pain relief was achieved with divided daily oral doses of 2 g. of paracetamol and 150 mg. of diclofenac sodium as well as 20 mg. of omeprazole per orally for the stomach protection. Full blood count, biochemistry, erythrocyte sedimentation rate (ESR), C-reactive protein (CRP) and urine analysis were obtained for all patients on admission. After the procedure all patients were evaluated according to the National Nasocomial Infection Surveillance (NNIS) System definitions for surgical site infections [7]. Body temperature, systemic blood pressure and pulse rates were recorded twice daily. White blood cell (WBC) count, ESR and CRP were repeated and samples were taken for cultures in the presence of any clinical evidence that suggests infection such as fever, infiltration in the lung parenchyma on CXR or turbid colour change of pleural drainage fluid. Chest tubes were removed 24 hours after cessation of the air leak and ensuing complete lung expansion on CXR. Air leak persisting for more than 7 days was accepted as "prolonged air leak". Those patients were considered for elective surgery, thus were excluded from the study. Drain stitches were removed as an outpatient on the 7th day following discharge from the hospital for all patients.

## Results

Of the 111 patients, there were 104 male and 7 female (M/F ratio = 15:1) with a mean age of 34.2 ± 9.7 (Range: 16 – 52) Patient's other demographic an clinical details are also shown in Table 1. Duration of the CTT was 2 days in 19 (17%), 3 days in 58 (53), 4 days in 29 (26%) and 5 days in 5 (4%) patients with a median of 3 days.

**Table 1:** Patient's Demographic Data and Clinical Findings.

	**n = 111**
**Mean Age ± SD (Min. – Max.)**	34,2 ± 9,7 (16 – 52)
**Gender (Male/Female)**	104/7 (15:1)
**Site of Pneumothorax (Left/Right)**	65/46
**Size of Pneumothorax (Partial/Total)**	85/26
**Duration of Tube Thoracostomy **(Median: 3 days)	**2 days: **19 (%17)
	**3 days: **58 (%53)
	**4 days: **29 (%26)
	**5 days: **5 (%4)

Local induration of skin around the entry site of the chest tube was observed in 28 (25%) patients and was attributed to local reaction of the foreign material and invasive procedure. None of them showed any systemic signs of infection, like fever, tachycardia or tachypnea. These patients were followed more closely without any intervention, and all of the local signs disappeared within two weeks time following removal of drain stitches without an additional treatment. In a subgroup analysis, WBC was found to be more than 11 × 10^3^.μL^-1 ^without an increase in either body temperature or serum CRP level in 12 (11%) patients and cultures from different sites did not show any significant growth. Therefore no antibiotics were started on these patients. On the other hand, 8 (%7) patients developed some degree of fever, not lasting for more than 48 hours. Their WBC and serum CRP levels were also within normal limits and cultures from suspected sites also remained sterile. None of the remaining 91 (82%) patients developed any clinical evidence of infection, therefore, neither routine blood tests were repeated nor were culture and susceptibility tests performed for these patients.

## Discussion

CTT is generally accepted as the first choice of treatment in the management of PSP. Other alternative treatment modalities such as oxygen inhalation with bed rest or simple aspiration using fine bore catheters [1] may be considered for patients with a small pneumothorax. Chemical pleurodesis after CTT or pleurectomy with the use of video-assisted thoracoscopy is recommended for recurrent episodes or even for the first episode in certain circumstances [8,9]. Patients who underwent CTT usually require hospitalization and their chest tubes are removed ensuing that air leak ceased and complete lung expansion has been achieved. Most patients are discharged home 24 hours after drain removal following a control CXR [5].

Wounds are classified in to 4 groups in surgical practice: clean, clean-contaminated, contaminated and dirty. As a surgical intervention, CTT is included in the "clean-contaminated" group of surgical wounds and infection risk of such a wound is 7.7% [10]. Although relevant reports are very limited in the literature, different opinions exist regarding the use of prophylactic antibiotics after CTT. An incidence of up to 6% of chest tube related empyema has been reported in trauma cases and suggested that the administration of prophylactic antibiotics should be considered, particularly where a prolonged period of chest tube drainage might be anticipated [11,12]. In an old controlled study [3] in which clindamycin was chosen for prophylaxis following CTT, the rates of pleural infection were 2,6% and 16% in the clindamycin and control groups, respectively. In contrast, prophylactic antibiotic use caused an increased morbidity in another study [4].

Patients with PSP are usually young adults with no systemic diseases and have enough resistance to bacterial infections. Because duration of CTT is less than a week and the procedure is also performed in a sterile environment, possibility of a bacterial infection is negligible in most cases. Thus, administration of a prophylactic antibiotic after CTT was omitted in patients who sustained PSP with no systemic disease during the study period of 3 years. We have also carefully monitored WBC counts and serum CRP levels as well as some clinical signs suggesting presence of an infection such as fever, tachycardia, and turbid appearance of the draining fluid and inflammation around the entry site of the chest tubes before making the diagnosis of an infection. Minimal inflammation and induration confined to the wound site have carefully been assessed in view of NNIS descriptions and were considered as foreign body reaction rather than a superficial infection. None of our patients developed any clinical signs (e.g. purulent discharge from the wound site, persisting fever, deterioration of the general condition, sepsis, pneumonia, empyema etc.) suggesting an infection that would necessitate an antibiotic treatment, and therefore supported our initial hypothesis. Following three and a half years of experience regarding this particular matter, we continue to employ the same policy with no increase in either infection or complication rates in our practice.

Unnecessary use of antibiotics has some consequences for both the individual patient and the institution regarding the cost-effectiveness and adverse reactions. Prophylactic antibiotic use has also been shown to have a critical role in the selection of antibiotic resistant bacteria to become the dominant colonizing flora and nasocomial pathogens for hospitalized patients [13]. Moreover, one would predict that increasing reliance on the most potent antibiotics used for prophylaxis will -in time- be associated with further increases in the prevalence of these antibiotic-resistant strains.

## Conclusion

Our study proved that the patients presenting with PSP do not require antibiotic prophylaxis during CTT. We believe in that such an approach not only decreases the cost of treatment but also prevents patients from undesired side effects of antibiotics.

## Authors' contributions

GO and CAK designed and coordinated the study and drafted the manuscript. UA carried out data collection and the literature search and also contributed to preparation of the manuscript. LM participated in the design of the study and the sequence alignment. She also helped to draft the manuscript. All authors read and approved the final manuscript.
